# Use of PCR-DGGE Based Molecular Methods to Analyse Microbial Community Diversity and Stability during the Thermophilic Stages of an ATAD Wastewater Sludge Treatment Process as an Aid to Performance Monitoring

**DOI:** 10.5402/2013/162645

**Published:** 2013-09-23

**Authors:** Anna V. Piterina, J. Tony Pembroke

**Affiliations:** Department of Chemical and Environmental Sciences and the Material and Surface Science Institute (MSSI), University of Limerick, Limerick, Ireland

## Abstract

PCR and PCR-DGGE techniques have been evaluated to monitor biodiversity indexes within an ATAD (autothermal thermophilic aerobic digestion) system treating domestic sludge for land spread, by examining microbial dynamics in response to elevated temperatures during treatment. The ATAD process utilises a thermophilic population to generate heat and operates at elevated pH due to degradation of sludge solids, thus allowing pasteurisation and stabilisation of the sludge. Genera-specific PCR revealed that Archaea, Eukarya and Fungi decline when the temperature reaches 59°C, while the bacterial lineage constitutes the dominant group at this stage. The bacterial community at the thermophilic stage, its similarity index to the feed material, and the species richness present were evaluated by PCR-DGGE. Parameters such as choice of molecular target (16S rDNA or *rpoB* genes), and electrophoresis condition, were optimised to maximise the resolution of the method for ATAD. Dynamic analysis of microbial communities was best observed utilising PCR-DGGE analysis of the V6-V8 region of 16S rDNA, while *rpoB* gene profiles were less informative. Unique thermophilic communities were shown to quickly adapt to process changes, and shown to be quite stable during the process. Such techniques may be used as a monitoring technique for process health and efficiency.

## 1. Introduction

Autothermal thermophilic aerobic digestion (ATAD), an advanced tertiary sludge treatment process, is used to produce a stabilized sludge (Class A biosolids) suitable for land spread [1–4]. ATAD treatment involves aeration in insulated jacketed reactors operating in a semibatch mode with temperatures ranging from 10°C at inlet to an average 65°C during the thermophilic stage. Microbial activity in the reactors, stimulated by aeration, results in the digestion of sludge solids, generating heat which is trapped in the insulated reactors, resulting in a rise in temperature (on occasion up to 70°C) and a pH rise to pH 9 as a result of ammonia generation from proteolysis. The combined activities of microbial degradation, alkalinity, and heat generation stabilize the sludge solids, while the heat results in pasteurization. Multiple factors, such as aeration, feed composition, feeding cycles, and operational parameters, determine the maximum temperature and time course achievable within the thermophilic stage [3, 5]. The sustainability of such a process is dependent on the long-term stability of the ecosystem and the activity of the microbial consortia within it, which in turn is highly dependent on ATAD design and operation [6]. When operating well, ATAD had been shown to remove enteric pathogens efficiently which is a prerequisite for its use as a fertilizer for land spread [7]. Therefore, measurement of the community structure, the diversity of species within the ecosystem, and the community dynamics as a function, of process stage and temperature are of critical importance as indicators not only of the health and stability of an ATAD process but for any wastewater treatment process [8, 9]. Diversity indices, which characterize an existing ecosystem, are known to be affected by perturbation in operational parameters and exposure to toxic or inhibitory substances or prevailing conditions, and they often correlate with nutrient limitations within the system. The rate of transition to extreme conditions can be important in allowing time for adaptation and succession to develop, while transition to extreme conditions can also result in lysis, denaturation of extracellular enzymes, inactivation of mesophilic microorganisms, the arrest of certain biodegradation process, and the initiation of others favored by the new conditions within the ATAD ecosystem. Thus, development and validation of biochemical and molecular methodologies to monitor and improve our understanding of the behavior of microbial communities within the ATAD system are required. Advances in molecular tools can allow unprecedented opportunities for detailed studies of species population structure and dynamics within biotechnological processes [10–12]. Determination of microbial abundance has often been a key focus accomplished by direct DNA extraction followed by PCR amplification of genera or domain-specific genetic biomarkers [13–16] and recovery of 16S rDNA sequences of Bacteria and Achaea and 18S rDNA of active fungi and eukaryotic organisms [17], while internal transcribed spacer (ITS) sequences within ribosomal operons are also informative [18]. Molecular fingerprints of diversity can be achieved by coupling PCR amplification of taxonomical targets with sequence dissimilarities analyzed by denaturing gradient gel electrophoresis (DGGE) enabling the separation of DNA fragments of identical length but of different sequence [19, 20], resulting in a DGGE fingerprint that is specific for the sample site. Although no standard tool exists, a variety of comparison tools are available to compare fingerprints [21] and include visual inspection, cluster analysis, nonmetric multidimensional scaling and moving window analysis, which allow several quantitative and qualitative biodiversity indices to be recovered. The Shannon diversity index (*H*′) is a quantitative index which computes band intensity with species abundance [22], while the estimation of species richness (*S*) provides qualitative data relating the total number of bands observed to the total species number and species richness (*S*) and corresponds to the total number of unique biomarker sequences within the ecosystem. PCR-DGGE is capable of detecting as much as 95–99% of the bacterial community, and several candidate sequences can be used as genetic biomarkers including variable regions within the 16S ribosomal DNA gene [23], the 23S ribosomal DNA gene, and the *rpoB* gene (encoding the *β* subunit of RNA polymerase) [24], which are universally present with highly conserved flanking regions allowing the simultaneous amplification with ‘‘universal primers” [25, 26]. Due to the length and sequence of polymorphisms, the choice of primers, their specificity and degree of mismatch, the reproducibility of extracting DNA from environmental samples with complex and diverse microbial communities and natural materials will affect the recovery of biodiversity indices [27–32]. PCR bias resulting from inhibition by coextracted impurities or preferential amplification of certain species by various primer pairs is also a key issue [31, 33–35]. Other important issues include the degree of genetic polymorphism within the target regions within the community [36], comigration of PCR fragments from different species in the same DGGE band [37], and the formation of multiple bands during amplification of genes from a single genome [38, 39]. Application of DGGE methodology also has size limitations of 100–500 bp, which may affect the probe design and further phylogenetic analyses, while subsequent quantitative analysis of the DGGE fingerprint obtained assumes that the number of bands reflects the microbial diversity in the sample [20, 21, 34] and that their relative intensity reflects abundance. The genomic copy number of 16S rDNA in certain species [40, 41], which can vary from one to more than 10 copies per cell [42], can also have a major effect [43, 44], while the occurrence of heteroduplex molecules [25] can effect interpretation. Hence, alternative biomarkers for PCR-DGGE fingerprints, such as the single copy *rpoB* gene [24], have been investigated, but because of amplification failure in certain strains [24] and limited information on primer specificity for *rpoB*, widespread application of this biomarker has been limited at least until more data on *rpoB* sequences is available. Thus, when utilizing PCR-DGGE to assess indicators of biodiversity and health of the ATAD process, understanding the key factors associated with the recovery of biodiversity is essential for optimizing the methodology.

## 2. Material and Methods

### 2.1. Sampling Site

Sampling was carried out at the municipal ATAD plant in Killarney, Co. Kerry, Ireland. Details of the plant, operating parameters, physicochemical characteristics for different stages of the ATAD process, pattern of organic matter transformation, and overall performance of the plant have been described [2, 30, 45, 46]. The daily feed rate was in the range of 15–30 m^3^·d^−1^ with the thickened sludge undergoing thermophilic digestion in a two-reactor (1A and 2A of 110 m^3^) semibatch process before the treated sludge was stored. Reactor 1A (the mesophilic reactor) operated between 35 and 49°C while Reactor 2A (the thermophilic reactor), operated between 58 and 65°C. 

### 2.2. Analytical Methods

Samples were taken at feed inlet (raw sludge untreated), from Reactor 1A, Reactor 2A, and from sludge storage (following treatment), in triplicate from the middle of each reactor via a sterile deep-water sampling device.

### 2.3. DNA Extraction

To enhance DNA extraction recovery and maximize diversity recovery, two extraction methods were applied in this study. A “Power Soil” DNA isolation kit (MOBIO, UK) was used as recommended by the manufacturer and another extraction protocol as described [31]. 

### 2.4. PCR Amplification

Oligonucleotides utilized in this study are detailed in Table 1. PCR primers amplifying different variable regions of the 16S rDNA (V3–V5 and V6–V8) and *rpoB* genes were used as described previously, except that PCR reaction contained 1% formamide (Sigma, UK) and 25 ng of nonacetylated BSA (Roche, UK) to limit inhibition of PCR reaction by impurities, originated from the thermophilic sludge [31]. To minimize PCR artefacts, the PCR amplification procedure was optimized using increased primer concentration to enhance the ratio of primer to template (30 pmol) [47], thus favoring primer annealing, and the amplification cycles were reduced from 30 to 20. Multiple (*N* = 10) PCR reaction mixtures for each DNA sample and for each PCR primer set were combined [40] and concentrated with two volumes of ethanol and glycogen [48]. Pellets were dissolved in 10 mM Tris-HCl (pH 8.0) to a final concentration 100 ng·*µ*l^−1^.

### 2.5. Denaturing Gradient Gel Electrophoresis (DGGE) Analysis

The PCR products were analyzed by denaturing gradient gel electrophoresis (DGGE) using the C.B.S. Scientific Co., Inc. (Del Mar, USA) system with integrated buffer and heating. Gel size was 22 × 22 cm and 0.75 mm thick with the electrophoresis buffer (0.5 X TAE) maintained at 60°C during electrophoresis. 

### 2.6. Preparation of a Reference Marker

To allow comparative analysis of DGGE patterns, a marker containing products amplified with primers from known bacterial species was used in each analysis. For this purpose, six bacterial strains, *Bacillus thuringiensis *NCIMB 9134 T, *Bacillus cereus *ATCC 14579, *Escherichia coli *JM 109, *Salmonella enterica *ATCC 29629, and two bacterial strains isolated and identified from ATAD sludge *Bacillus subtilis *ATAD 1 and* Bacillus licheniformis *ATAD 6, were routinely used. A colony of each isolate was suspended in 50 *µ*L lysis buffer (0.05% SDS; 0.03 M NaOH) and incubated for 15 min at 95°C. 450 *µ*L of distilled water was added and centrifuged for 5 min at 13,000x rpm, and 1 *µ*L of each supernatant was used for PCR. The migration behavior of each of the amplicons was analyzed via DGGE, and those showing different migration distances on the gel were combined and used as a reference lane. Marker samples were stored at −80°C.

### 2.7. Optimizing the DGGE Protocol for ATAD Samples

*(a) Sample Electrophoresis Time. *Optimal duration of electrophoresis and migration via time course experiments was evaluated to obtain maximum resolution for samples from ATAD Reactors 1A and 2A and product following processing. 500 ng of PCR amplicon was subjected to electrophoresis for 1, 2, 3, 10, 16, 18, and 19 h at 75 V and 250 V, with buffer temperature maintained at 60°C on 8% or 10% PAAG for *rpoB* fragment amplicons, 1, 2, 3, 10, 16, 18, and 19 h at 75 V and 250 V on 6% or 8% PAAG for V6–V8 amplicons, and 8% or 10% for V3–V5 amplicons of 16S rDNA.

*(b) Optimization of Sample Loading to Maximize DGGE Band Recovery. *The detection limit of bands achievable by DGGE analysis is variable, but optimization is essential to maximize biodiversity index recovery from a DGGE fingerprint. The amount loaded can depend on the total number of different amplicons, their copy number, and the species diversity and richness present, factors that are often impossible to predict prior to analysis. Thus, the amount of PCR product loaded on gels was varied within the range of 100 ng to 5 *µ*g. Gels giving the maximum banding patterns were chosen for comparative DGGE analysis. At least two gels using the same conditions were routinely used for such comparative analysis. Gels were stained with either silver stain [49] or ethidium bromide [48].

*(c) Analysis and Comparison of DGGE Patterns. *DGGE profiles were analyzed for similarities via digital image analysis using the BioNumerics 4.0 software package. DGGE bands were detected using the band-search algorithm and following background subtraction; profiles were normalized using a species standard as a reference. Only those bands with a peak height intensity exceeding 1.0% of the strongest band in each lane were included for further analyses. A mathematical diversity index (complexity richness (*S*)) was calculated for each PCR-DGGE fingerprint generated from sludge samples taken at different stages of ATAD treatment by the software-based enumeration of the total number of bands in each lane as well the number of unique bands in the DGGE profile obtained for thermophilic sludge DNA. The % similarity between the two fingerprints was calculated based on the number of common bands between two fingerprints. The % change value matrix was used to perform moving window analysis by plotting the values between two consecutive sampling points:

(1) %  changes=100−%  similarity

(see [21]). Consecutively, the rate of change (*D*_*t*_) of overall community dynamic was calculated according to the average of the respective moving window curve data points:

(2) Dt=∑N(%  changes)=Δt

(see [21]). The bigger the change between DGGE profiles from two consecutive sampling points, is the higher the corresponding moving window curve data point will be and, hence, the higher the *D*_*t*_ values. A range-weighted richness (Rr) index can be mathematically expressed by defining the total number of bands observed multiplied by the percentage of denaturing gradient needed to generate the total diversity of the sample. This value describes a carrying capacity of an environment containing wide species GC variability (both in terms of percentage and in terms of positioning of the GC stretches within the 16S rDNA gene). This value was characterized according to the following formula:

(3) $$\begin{array}{c} \text{Rr}=N^{2}\times D_{g} \end{array}$$

(see [21]), where *N* represents the total number of bands in the pattern and *D*_*g*_ the denaturing gradient occurring between the first and the last band of the pattern analyzed. Rr (richness) was determined from fingerprints for each type of sludge sampled during the ATAD process and for each experimental setting. Similarities between DGGE patterns were calculated using band presence or absence via pairwise similarity of the banding patterns for the different samples, and clustering of patterns was calculated using the unweighted pair group method using average linkages (UPGMA) to determine whether the samples revealed a nonrandom pattern and whether they clustered according to specific patterns (temperature in the reactors, time of operation within the same reactor, or aeration effects). It was hypothesized that important environmental stressors may be determined by the application of such methods. 

## 3. Results

### 3.1. PCR Survey of the Biodiversity of the ATAD Ecosystem

High-molecular-weight total genomic DNA extracts were prepared from sludge samples taken during the ATAD process by a combination of two direct DNA extraction methodologies to maximize DNA template recovery as recommended previously [31, 32]. A PCR survey of the recovered DNA templates using a variety of genera-specific primers (Table 2) was performed. Results revealed that the ATAD system is dynamic and undergoes decline in the number of lineages as the treatment proceeds, possibly due to increased pH and temperature within the ATAD process. Archaeal lineages (Table 3), although detected in the feed, were below detection limits at later stages of the ATAD process. Fungi were also detected in the feed sludge and within the mesophilic sludge (Reactor 1A) and were detected again within the stored product but were not detected in the thermophilic reactor 2A. Eukaryotic sequences were also not detected in the thermophilic ATAD reactor. Genomic DNA from bacterial lineages was detected at all stages of the ATAD treatment, with only bacterial species being detected at the thermophilic stage (Reactor 2A).

### 3.2. DGGE Fingerprinting of Bacterial Community Diversity

Based on the initial PCR survey, the major driving force for the elevated temperature and the biodegradation occurring at elevated temperature within the ATAD process would appear to be bacteria. To determine the nature of the bacterial populations supported at each stage of ATAD, we utilized PCR-DGGE profiling techniques. PCR fragments for well-known taxonomical biomarkers such as V3–V5 and V6–V8 regions of the 16S rDNA and the *rpoB* gene were amplified from the DNA template, recovered from ATAD, and analyzed following optimization of template extraction, template amplification, and DGGE separation. By optimization of the DGGE protocol, it was possible to maximize band recovery for all types of DNA profiles (of mesophilic and thermophilic origin) analyzed which resulted in excellent band separation, detection, and visualization (Table 4). Comparison of the DGGE band patterns sampled from the same ATAD stage showed a high degree of reproducibility both between PCR amplifications, where the same DNA extract was amplified multiple times, and between DGGE gels, where the same PCR product was loaded several times.

**Figure 1:**
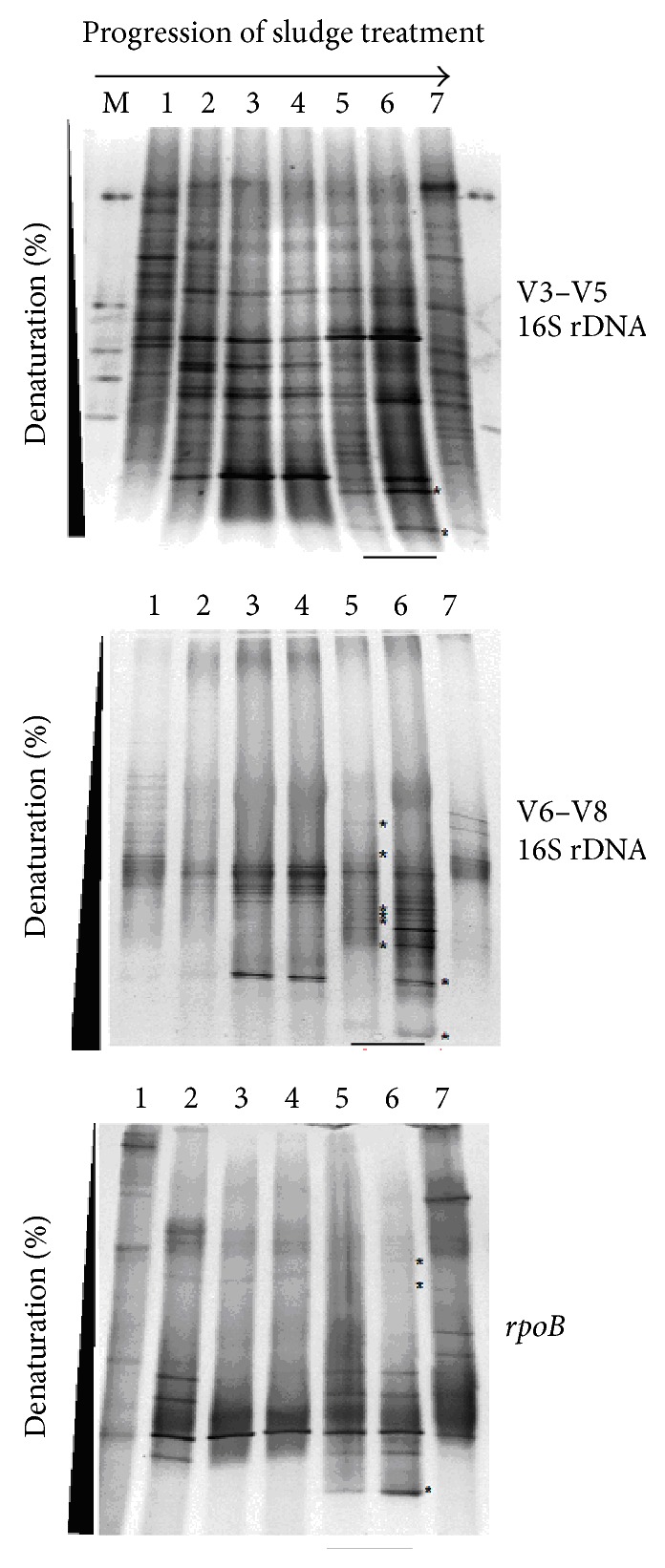
Succession of bacterial communities during the ATAD process as monitored by DGGE fingerprints from DNA targets amplified by PCR from directly extracted ATAD sludge DNA. Primers targeted conserved regions of the V3–V5 and V6–V8 regions of the bacterial 16S rDNA and *rpoB* gene sequences. Lane 1: thickened sludge, Lane 2: Reactor 1A (after 4 hours), Lane 3: Reactor 1A (after 8 hours), Lane 4: Reactor 1A (after 16 hours), Lane 5: Reactor 2A (after 4 hours), Lane 6: Reactor 2A (after 23 hours),and Lane 7: product (biosolids after treatment). The pattern for thermophilic sludge (Reactor 2A) is illustrated by the underlying arrows. Unique bands from the thermophilic fingerprints are highlighted with (∗) on the right side of the lane. The bar indicates the profile from the thermophilic stage in Reactor 2A.

Optimized conditions were utilized to profile heterogeneity within the total pool of amplified V3–V5 and V6–V8 amplicons of the 16S rDNA and the *rpoB* gene from templates derived from various ATAD samples, and then, these DGGE fingerprints and band patterns were compared and evaluated. The patterns recovered indicated that the ATAD process is a dynamic ecosystem (Figure 1). Shifts in bacterial composition at different times during the ATAD treatment were observed for patterns produced by the three taxonomic genetic markers used. Many bands present at the autoheating stage (Reactor 1A) were absent from the DGGE pattern at a later stage in the ATAD process (Reactor 2A) indicative of succession and adaptation of new species to the new process conditions and the replacement of species that perhaps cannot adapt. In the DGGE community fingerprint of 3 biomarkers obtained from Reactor 2A, a new DGGE band pattern could be observed indicating the development or growth of new bacterial species under these operational parameters. The new bands observed in the Reactor 2A DGGE fingerprint (Figure 1) for all 3 biomarkers migrate further in the gel indicating a highermelting temperature for these DNA amplicons than for those recovered from mesophilic sludges (Reactor 1A, inlet, product) which may be explained by an increased CG content of these amplicons. The DGGE patterns suggest that as the ATAD process proceeds, new bands appear (Figure 1, Lanes 6 and 7) indicative of new species emerging, while certain bands present in the mesophilic sludge samples are also retained indicating that certain species have adapted to the elevated temperature and pH conditions in Reactor 2A.

**Figure 2:**
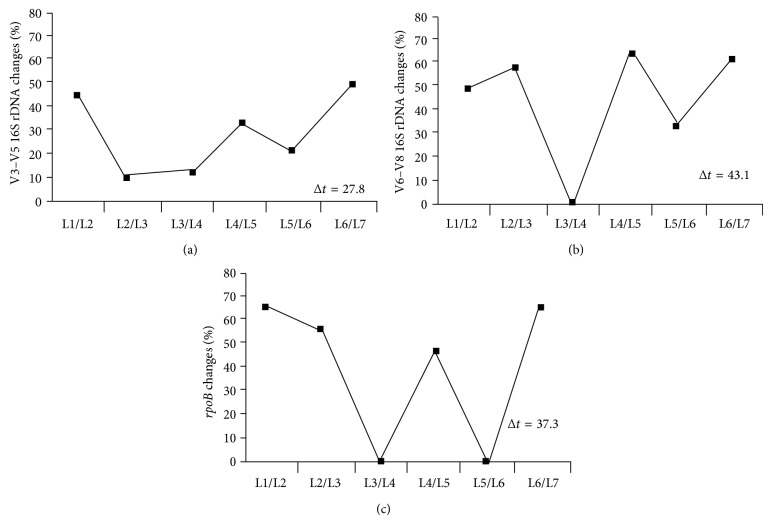
Moving window analyses (MWA) and rate of change (Δ*t*) values to evaluate the level of dynamics of the bacterial community during ATAD treatment. Primer sets were targeted to the V3–V5 and V6–V8 regions of the bacterial 16S rDNA and *rpoB* gene sequences. L1 thickened sludge, L2 Reactor 1A (after 4 hours), L3 Reactor 1A (after 8 hours), L4 Reactor 1A (after 16 hours), L5 Reactor 2A (after 4 hours), L6 Reactor 2A (after 23 hours), and L7 product (biosolids after treatment). Values were calculated as described in Section 2.

Overall we observed a significant difference between the total microbial communities of influent and thermophilic reactors. The number of bands obtained from thermophilic samples for primers specific to the V3–V5 region of 16S rDNA was higher than that for the V6–V8 region or for *rpoB* gene (10 as opposed to 22 bands). Dendrograms created for the V6–V8 region showed that the microbial community in the thermophilic reactor at 4 hours and 24 hours can be grouped into distinct clusters (Figure 2) 11 new DGGE bands were observed, while for the *rpoB* amplicon pool and only two new bands were observed. The numbers of unique bands obtained for the thermophilic stage of ATAD were quantified using the BioNumerics 4.0 software, summarized in Table 3. The number of DGGE patterns obtained for the V3–V5 region for the thermophilic stage was highest with fewer bands observed from the V6–V8 region. This may reflect less variability in amplicons originated from this region resulting in fewer different bands. 

**Figure 3:**
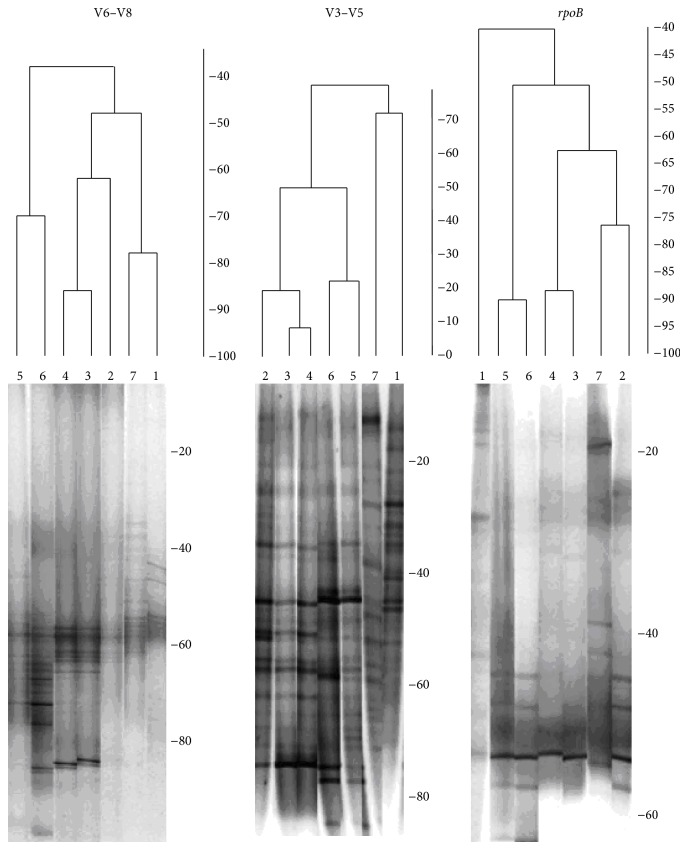
UPGMA dendrogram of DGGE profiles of amplified 16S rDNA of the total bacterial community from ATAD sludge obtained at different stages of the ATAD process using BioNumerics 4.0 software advanced analysis package. The fingerprints were obtained with primers amplifing the V6–V8 and V3–V5 regions of 16S rDNA and the *rpoB* gene. The cluster tree images are aligned alongside. Lane 1: thickened sludge, Lane 2: Reactor 1A (after 4 hours), Lane 3: Reactor 1A (after 8 hours), Lane 4: Reactor 1A (after 16 hours), Lane 5: Reactor 2A (after 4 hours), Lane 6: Reactor 2A (after 23 hours),and Lane 7: product (biosolids after treatment).

The application of moving window analysis, where the differences between profiles were plotted as a function of ATAD process steps (Figure 2), revealed a clear shift in bacterial population richness with the number of dominant and recessive bands for each biomarker varying. Changes in the richness (Rr) within the population as a function of the ATAD treatment steps could be detected for all the primers used (Figure 3). After 16 hours of processing in Reactor 1A, there was a decline in population richness. Numerical comparison of the DGGE fingerprints for the V6–V8 region 16S rDNA amplicons at 4 hours and 23 hours in Reactor 2A provides evidence that the community richness is increased, indicating that 4 hours after the feeding within Reactor 2A, only a few specialized, adapted, and possibly less diverse microbial species are predominant, whereas 23 hours after feeding, the prevailing conditions may support activity and growth of more diverse bacterial species (more diverse DGGE profile). These observations indicate that caution is necessary in the choice of amplicons used in studying such population dynamics. The similarities between the DGGE patterns produced from fingerprints of the various amplicons for each reactor were calculated and visualized as a clustered tree to evaluate the dynamics within the groups of bacteria (Figure 3). Clear differences in bacterial community structure were observed from each point sampled during the ATAD process, which is consistent with suggestions that the process temperature is the driving force for bacterial selection and adaptation. However, dendrograms derived from DGGE fingerprints, for the V3–V5 region indicate that the fingerprint of microorganisms from the thermophilic stage are clustered together with the profile of the community from the mesophilic sludge (Reactor 1A) (Figure 3). This may indicate that the communities are similar or related to a degree and share common bacterial members. The recovered richness Rr indexes for DGGE fingerprints for each population at different stages of ATAD were found to be influenced by the primer set used for the analysis (Table 4). In the case of community data recovered from thermophilic Reactor 2A, the greatest richness value was obtained using primers for the V3–V5 region of 16S rDNA (Rr = 113.9–104.4) and the lowest observed for primers for the *rpoB* gene (Rr = 9.1–13.6). In comparison to other studies (reviewed in [21]), the richness of the ATAD thermophilic community (Reactor 2A) was less than that for soil ecosystems, which are known to be rich in the number of bacterial lineages, but in a similar range to that observed in the activated sludge [21] This data supports the suggestion that the ATAD thermophilic process presents a selective environment supporting a specialized bacterial community. 

## 4. Discussion

The diversity of wastewater microorganisms is an important ecological parameter, which has been mainly expressed using ecological indices such as the Shannon or Simpson indices [50]. While the community diversity and composition are important, the dynamics of microbial communities are also a key issue, as this plays an important role in the functionality and health of a system. Generally, examination of dynamics and perturbation of functionally stable microbial communities has been overlooked in studies of ecosystems and especially wastewater treatment systems. Previous studies on ATAD have focused on the microbial populations at the thermophilic stage [1, 32, 51] with little attention being paid to how the populations change as a function of process progress and, indeed how, this could be monitored. 

Here DGGE of PCR-amplified 16S rDNA and* rpoB *gene fragments from samples taken from an ATAD treating domestic waste at full scale revealed an active interaction between the physicochemical parameters during the process and the bacterial community. This is consistent with analogous levels of bacterial community dynamics in bench-scale thermophilic digestion systems and full-scale treatment systems applied for biodegradation of pharmaceutical waste [1]. Data indicated that the microbial community had a mesophilic origin in the primary and secondary sludge and subsequently adapted to the changing environmental conditions of the ATAD process at elevated temperature and high pH during the thermophilic stage. This adaptation of the bacterial community leads to a stable process resulting in the pasteurization of ATAD sludge at the thermophilic stage. This adaptive capacity of the bacterial community is a key factor in the overall ATAD process. One of the aims of this study was to determine whether the combination of parameters, such as pH, oxygen availability, and the temperature regime in the ATAD process reactors, would serve as a driving force for the selection of specific active microflora within each reactor. Using PCR-DGGE techniques, we initially evaluated the effect of the methodology on the reliability of the analysis. 

A key initial factor was the ability to extract total DNA from the ATAD system such that maximum recovery of the total microbial population would be achieved. Maximum recovery could be achieved by utilizing multiple extraction methods and pooling samples for analysis [31]. There have been many reports of conflicting estimates of microbial diversity due to the extraction protocols [31, 52–54], but there have been few applications of multiple extraction techniques and pooling to address the issue. A touchdown PCR technique was applied for amplification [55] using different sets of primers, as such techniques have been considered useful to avoid the amplification of spurious DNA fragments (non-rDNA fragments and/or fragments of improper size) [55].

Kingdom specific probes were initially employed and detected fungal sequences at inlet and at the early stages within mesophilic Reactor 1A. Fungal sequences were not detected at later stages in Reactor 1A, which can be characterized by a pH rise between pH 8.3 and 9 and a temperature rise to 53–55°C (Table 2). The alkaline pH of 8.3–9 and the high temperature are not supportive environments for Eukarya or Fungal species and are possible reasons why neither was detected at the thermophilic stage. Aeration in Reactor 1A and Reactor 2A is probably a factor responsible for the inability to detect Archaeal species although these were present at inlet. Bacterial species were detected at all stages and were in fact the dominant kingdom in thermophilic Reactor 2A.

Although there are numerous reports [28, 56–59] of using PCR-DGGE to examine microbial diversity, the quality of the information obtained is impacted by the choice of probe, specifically the type of gene used for analysis or that if using the 16S rDNA gene, the V region(s) chosen. Comparison of nucleotide sequences has shown that there are regions of rDNA sequences that are highly conserved between all organisms and other regions that vary to different degrees. The variability in these regions increases as the evolutionary distance between two organisms increases, which provides a means to determine phylogenetic relationships and to distinguish microorganisms from one another [60]. Here, three primer sets were chosen because they have been used in a number of studies for the characterization of microbial communities [20, 39, 61]. For thermophilic aerobic ecosystems such as ATAD little previous information was available hence, two V-region-specific primers to the 16S rDNA gene and primers to the *rpoB* gene were examined. The results presented on the ATAD (Figure 2 and Table 4) sludge community DNA samples indicated that the genetic template chosen for amplification and analysis by DGGE can influence the recovery of richness and diversity indices, as well as the ability of the DGGE method to detect the changes within the community. The degree of variability of the chosen genetic marker within the community and the degree of dissimilarity between the nucleotide sequences of bacterial members inhabiting ATAD sludge at various treatment stages are the most decisive factors in determining the success of ecological monitoring (Figure 1). Another factor that needs optimization is the separation parameters within DGGE, which include gradient range and electrophoresis parameters such as voltage and time. Such parameters have received limited attention for mesophilic communities, and no data is available for parameter optimization for thermophilic bacterial communities where the GC content of DNA increases and can potentially affect DGGE fingerprinting methods. Three different primer sets were utilized to amplify nonoverlapping regions of the 16S rDNA gene V3 to V5 (set A), V6–V8 (set B) and the *rpoB* gene (set C). PCR amplification and DGGE fingerprint analysis of the ATAD samples via V3 to V5 region and V6–V8 region produced superior dynamic profiles with V6–V8 profiles being easier to compare between sampling sites. Variations (Figure 2) within the DGGE fingerprint for V3–V5 and V6–V8 amplicons were observed at sampling sites, such as Reactor 2A at 4 and 23 hours, indicating the possible development of microcosms with differing predominant species and diversity indicative of temperature, substrate [30], and oxygen solubility changes within the thermophilic reactor. Primer sets for the V6–V8 region produced the highest number of thermophilic phylotypes, whereas the V3–V5 region primers resulted in the recovery of the highest number of phylotypes from the less thermophilic environments at earlier stages of the ATAD process. This suggested that the latter pool may be more genetically variable, and pattern differences may be expected because GC content and nucleotide conservation are not constant for the length of the gene [39, 62]. The fingerprints obtained by the DGGE method were used to examine the similarity of a group of samples taken at different process stages and calculated from binary matrices (Figure 3). As the intensity of DGGE bands can be or influenced by small PCR errors or the DNA extraction efficiency of rare templates, the analysis of intensity matrices during comparison was not utilized. The choice of fragments used for amplification and DGGE profiling influenced how profiles were grouped together and the degree of similarity observed between ATAD sludge samples. The V6–V8 pool (setB) of 16S rDNA amplicons was able to cluster the community not only in relation to the changes in temperature parameters, but in correlating nutrient availability and stage of treatment (Figure 3, Table 4). Set A primers (V3–V5 region) amplified a smaller 16S rDNA fragment which contained more sequence variability within the microbial community and was more useful in profiling variability within the mesophilic sludge (Reactor 1A). In the thermophilic sludge, set B (V6–V8) primers gave more discriminating profiles. These data suggested that set B (V6–V8) primers would be more useful for further ecological monitoring and analysis of the thermophilic microbial communities.

It has been proposed [21] that in addition to standard DGGE gel comparison, additional statistical analyses could be applied for comparative purposes. A statistical approach was utilized in comparing ATAD microbial communities using a moving window correlation (Figure 2). The principle of moving window correlation, a more quantitative measure to evaluate diversity and evolutionary shifts over processing time, can generally be used to monitor the effect of process evolution and changes in ecological parameters on population variability within or between systems. This can be used to define a stability rate for the microbial community under investigation, and as opposed to principal component analysis, multidimensional scaling, cluster analysis, and diversity indices, this technique includes a time-based analysis of the DGGE data. The capacity of this DGGE analysis to monitor the changes and stabilization of the microbial community at various ATAD treatment steps over time was evaluated by comparing the results obtained with the measured variability of DGGE community profiles obtained with various genetic biomarkers (Figure 2). This latter method was based on the within-gel variability of DGGE analysis obtained for each verified pool of three biomarkers and a stability criterion applied to evaluate the results. Based on the coefficients obtained, the stability profiles for the different reactors and primer sets were quantitatively evaluated and compared to each other. A clear change in population richness was obtained for each biomarker however, the degree of change was variable for each marker and different markers showed variable utilities depending on the process stage and whether it was thermophilic or not (Table 4). The *rpoB* marker appeared to be least informative during our ATAD analysis (Table 5). Changes in richness were also monitored as proposed [21] and indicated (Figure 2) that the V3–V5 marker was most informative at the mesophilic/thermophilic interface, whereas the V6–V8 was more useful during the thermophilic stage between 4 and 23 hrs of thermophilic treatment. Primer sets for the V6–V8 region produced the highest number of thermophilic phylotypes, whereas the V3–V5 region primers resulted in recovery of the highest number of phylotypes from the less thermophilic environments at earlier stages of the ATAD process (Table 5). This suggested that the latter pool may be more genetically variable, and pattern differences may be expected because GC content and nucleotide conservation are not constant for the length of the gene [39, 62]. Therefore, unless the proper primer sets are determined for DGGE monitoring at thermophilic stages, errors in interpretation and conclusions can be made in relation to the diversity present, and this may indeed be a principle when DGGE is used in process monitoring in general. Here, the V6–V8 analysis indicated the presence of increased diversity at the thermophilic stage and not a decline in diversity as previously reported for ATAD treating pharmaceutical waste [1]. Analysis of the sludge composition in Reactor 2A (using V6–V8 primers) clearly indicated an interaction between the physicochemical features of the reactors and the microbial community present and suggested that depletion of nutrients and carbon sources may serve as a dominant factor for population changes at later stages of ATAD thermophilic treatment [30, 46]. These data provide support for the hypothesis [51] that temperature is not the only factor acting as an environmental stressor in ATAD wastewater treatment systems and suggest that the thermophilic community is highly adaptable to changes in process conditions and sludge characteristics. This analysis highlights previous concerns [28, 57, 58] that results of DGGE-based community structure analyses should be interpreted cautiously and experimental settings for new environmental samples need to be optimized carefully. The elevated GC content of DNA in thermophilic organisms is known as an adaptive mechanism to increased temperatures, and this is observed in the patterns obtained from the thermophilic stages of the ATAD process. DGGE, once optimized, is thus a promising way of investigating bacterial community structure and dynamics within aerobic thermophilic communities and is an aid to monitoring process health. However, as only partial sequences are generated from DGGE bands, this may limit taxonomical and species assignment. Unlike the 16S rDNA gene, where multiple copies can exist within genomes which complicate diversity assessment, the gene for the beta subunit of the RNA polymerase, *rpoB*, is a single copy gene and offers advantages on occasion. When the *rpoB* gene was amplified, fewer DGGE bands were observed which may be reflective of the close GC content of the microbial population in Reactor 2A or the close sequence similarity of this select population. In either event, its use was less discriminating than the 16S rDNA primers for aerobic thermophilic populations. 

## 5. Conclusion

The overall aim of the ATAD process is to generate a pasteurized, stabilized sludge suitable for classification as a Class A biosolid. Given that the driving force is heat generated by a thermophilic biodegradative population, systems such as PCR-DGGE may prove useful in monitoring the optimal characteristics of the system. The protocols described here, upon optimization, can easily be applied for routine monitoring of bacterial populations within thermophilic niches such as ATAD waste treatment and can be of use in monitoring the process health and indeed stability of the process as a function of time. Use of 16S rDNA sequencing of excised DGGE bands can prove useful for further phylogenetic analyses of such ATAD systems, and these studies are underway [32].

## Figures and Tables

**Table 1:** List of the oligonucleotide primers utilized in this study.

Primer		Sequence (5′-3′)^2^	Specificity	Techniques	Reference
Taxonomic lineage	Name of primer				
Bacteria	27F^1^	AGAGTTTGATCCTGGCTCAG	V1, 16S rDNA	PCR	[63]
	1492R^1^	GGTTACCTTGTTACGACTT	V9, 16S rDNA	PCR	[63]
	GC-338F	ACTCCTACGGGAGG CAGCAG	16S rDNA	PCR, DGGE	[19]
	518R	ATTACCGCGGCTGCTGG	16S rDNA	PCR, DGGE	[19]
	GC-948F	AACGCGGAAGAACCTTAC	V6, 16S rDNA	PCR, DGGE	[39]
	L1401R	CGGTGTGTACAAGAAGACCC	V8, 16S rDNA	PCR, DGGE	[63]
	GC-rpoB1698F3	AACATCGGTTTGATCAAC	rpoB	PCR, DGGE	[24]
	rpoB2041R	CGTTGCATGTTGGTACCCAT	rpoB	PCR, DGGE	[24]

Fungi	F3F	TCCTCTAAATGACCAAGTTTG	18S rRNA	PCR	[13]
	EF4R	GGAAGGG[G/A]TGTATTTATTAG	18S rRNA	PCR	[13]

Archaea	Arc 21F	TTCCGGTTGATCCYGCCGGA	16S rRNA	PCR	[64]
	Arc 958R	YCCGGCGTTGAMTCCAATT	16S rRNA	PCR	[64]

Eukarya	Euk1427F	TCTGTGATGCCCTTAGATGTTCTGGG	18S rRNA	PCR	[65]
	Euk1616R	GCGGTGTGTACAAAGGGCAGGG	18S rRNA	PCR	[65]

^1^F: forward primer; R: reverse primer. The numbering denotes positions of primers relative to the *E. coli* 16S rDNA gene.

^2^GC clamp added to the 5′ end of the primer 338, 5′CGCCCGCCGCGCGCGGCGGGCGGGGCGGGGGCACGGGGG G 3′.

Sequences represent the nucleotide sequences in the GC clamps of the respective primers.

**Table 2:** Detection of microbial diversity in ATAD sludge at different stages of ATAD treatment. PCR amplification using Archaea-, Eukarya-, Bacteria-, and Fungal-specific primers as listed in Table 1 was used.

Origin of the extracted DNA	PCR amplification with genera- and domain-specific primers			
	Eukarya	Archaea	Bacteria	Fungi
Inlet type I (sewage)	+	+	+	+
Inlet type II (secondary sludge)	+	−	+	+
Thickened inlet sludge	+	+	+	+
Reactor 1A ATAD (2 hours of operation)	+	−	+	+
Reactor 1A ATAD (4 hours of operation)	−	−	+	+
Reactor 1A ATAD (16 hours of operation)	−	−	+	−
Rector 2A ATAD (4 hours of operation)	−	−	+	−
Reactor 2A ATAD (23 hours of operation)	−	−	+	−
Biosolids (9 days and storage)	+	−	+	+

Positive detection is indicated as (+) ve whereas lack of amplification is indicated as (−) ve.

**Table 3:** Optimized conditions for DGGE analysis.

Amplicon	Primers set	Optimal parameters (DGGE analysis)					
		Amount of DNA template in PCR reaction	Voltage, V	Time, hrs	Denaturant range, (%)	Amount of amplicon (per lane), *μ*g	Gel staining methods
V3–V5; 16S rRNA	338F-GC^*^/518R	300 ng	75	16	35–75	4.5	EtBr
V6–V8, 16S rRNA	948F-GC^*^/L1401	300 ng	75	18	40–65	1.5 3	Silver stain EtBr
*rpoB *	1698F-GC^*^/2043R	600 ng	75	16	30–60	0.3	EtBr, silver stain

^*^GC clamp added to the 5′ end of the primers 338F, 948F, and 1698F, 5′CGCCCGCCGCGCGCGGCG GGCGGGGCGGGGGCACGGGGGG-3′.

**Table 4:** Comparison of range-weighted richness (Rr) for bacterial diversity at different stages of ATAD treatment obtained by various primer sets in this study and values reported in other studies from different industrial and native ecosystems. Rr was calculated for each DGGE profile obtained as described in Section 2. The highest Rr values are shown in bold.

Fingerprint type	Range-weighted richness, Rr			
	16S rDNA		*rpoB *	Reference
	V3–V5 region	V6–V8 region		
ATAD inlet	**80.2**	**50.4**	**56.40**	This study
ATAD Reactor 1A (after 4 hours)	**50**	1.53	36.56	This study
ATAD Reactor 1A (after 8 hours)	**40.5**	12.67	4.05	This study
ATAD Reactor 1A (after 16 hours)	6.61	**12.67**	4.05	This study
Reactor 2A (after 4 hours)	**140**	15.9	7.2	This study
Reactor 2A (after 23 hours)	**113.9**	**62.5**	9.1	This study
Biosolids (after post treatment)	**104.4**	12.1	13.6	This study
Pharmaceutical activated sludge	25	—	—	[21]
Stable nitrifying reactor	**145**			[21]
Municipal activated sludge	57	—	—	[21]
Garden soil	**220**	—	—	[21]
Legume rhizosphere	57	—	—	[21]
Arctic sea ice	26	—	—	[21]

**Table 5:** Comparison of the efficiency of different primer sets to monitor diversity of the bacterial community inhabiting the elevated temperature reactor (ATAD Reactor 2A) at various times of reactor operation (after 4 hours and 23 hours).

Genetic biomarker	DGGE profile analysis of PCR product				
	Diversity^a^		Number unique band		Total numbers of the unique bands appearing during operation of Reactor 2A
	Reactor 2 (4 hrs)	Reactor 2 (23 hrs)	Reactor 2 (4 hrs)	Reactor 2 (23 hrs)	
16S rRNA gene					
V3–V5	19	18	5	2	7
V6–V8	14	12	5	6	11
*rpoB *	4	6	1	1	2

^a^Total number of bands detected on a DGGE lane.
